# Synthesis of Cellulose-Based Hydrogel—Nanocomposites for Medical Applications

**DOI:** 10.3390/polym16152183

**Published:** 2024-07-31

**Authors:** Wala`a Al-Tarawneh, Imad Hamadneh, Ola Tarawneh, Ali Al Najdawi

**Affiliations:** 1 Department of Chemistry, University of Jordan, Amman 11940, Jordan; walla_chem@yahoo.com; 2 Department of Pharmacy, Faculty of Pharmacy, Al-Zaytoonah University, Amman 11733, Jordan; ola.tarawneh@zuj.edu.jo; 3 Department of Biomedical and Chemical Engineering, University of Texas at San Antonio, San Antonio, TX 78249, USA; ali.alnajdawi@my.utsa.edu

**Keywords:** hydrogel, cellulose, carboxy methyl cellulose, nanocomposite, yttrium oxide (Y_2_O_3_)

## Abstract

This study focused on synthesizing a cellulose-based hydrogel nanocomposite as a green hydrogel by adding a microcrystalline cellulose (MC) solution to carboxymethyl cellulose sodium (CMC-Na) with citric acid as a cross-linker. Y_2_O_3_ nanoparticles were incorporated during hydrogel preparation in different ratios (0.00% (0 mmol), 0.03% (0.017 mmol), 0.07% (0.04 mmol) and 0.10% (0.44 mmol)). FTIR analysis confirmed the cross-linking reaction, while XRD analysis revealed the hydrogels’ amorphous nature and identified sodium citrate crystals formed from the reaction between citric acid and CMC-Na. The swelling test in deionized water (pH 6.5) at 25 °C showed a maximum swelling percentage of 150% after 24 h in the highest nanoparticle ratio. The resulting cellulose hydrogels were flexible and exhibited significant antibacterial activity against *Staphylococcus aureus* (*S. aureus*) and *Escherichia coli* (*E. coli*). The synthesized cellulose-based hydrogel nanocomposites are eco-friendly and suitable for medical applications.

## 1. Introduction

The development of advanced biomaterials for medical applications has earned significant attention, driven by the growing need for innovative solutions to address complex healthcare challenges [1]. Hydrogels are materials with a three-dimensional network of hydrophilic polymer groups that have the unique ability to retain substantial amounts of water [2]. This unique structure grants hydrogels beneficial properties such as biocompatibility, exceptional hydrophilicity, and a high capacity for water absorption. As a result, hydrogels find applications in a wide range of fields, including but not limited to ionic skin, tissue engineering, controlled drug delivery and release, and wound dressing and healing. This versatility in applications underscores the potential of hydrogels in various industries [3,4].

Also, the efficiency of hydrogel properties justifies its widespread applications and improvement in recent technology where the process becomes inventive to suit specific desired functions. The most investigated naturally occurring hydrogel polymers are cellulose and cellulose derivatives. Cellulose, a naturally abundant polysaccharide found in plant cell walls, has long been recognized for its biodegradability, low toxicity, and renewable nature, making it an appropriate substrate for biomedical applications [5]. The cellulose derivative hydrogel containing poly-L-lysine and tea polyphenols speeds up the healing of infected wounds and prevents bacterial infection [6,7].

The cellulose hydrogel loaded with chlorhexidine has shown a promising local delivery system for treating periodontitis [8]. The synthesis of cellulose derivative biofilm prepared from hydroxyethyl cellulose (HEC) and Na-CMC loaded with nystatin (NYS) exhibited high glassy temperature, T*_g_* of 131.79 ± 2.86 °C, high tensile strength of 4.25 ± 0.53 GPa, high mucoadhesion properties of 5.12 ± 0.20 N/mm^2^, and the ability to release the drug within 3 to 8 h [9].Cellulose/nanocomposite hydrogels are a mixture of natural cellulose and/or cellulose derivative hydrogel and nanostructured materials have emerged as promising candidates due to their unique properties, biocompatibility, and versatile applications in various medical fields [3,4,5,6,7].

The scaffold hydrogel prepared from collagen/nanocrystalline and cellulose/chitosan loaded with Au NPs was studied by Nezhad-Mokhtari et al., 2020 [10]. The result showed an improvement in mechanical strength and degradation resistance without any noticeable cytotoxicity for 3T3 fibroblast cell lines [10]. The introduction of gold nanoparticles into polymeric hydrogel showed a great healing effect in rats and could be employed in wound healing [11].

Furthermore, the integration of nanoparticles into cellulose derivatives further enhances their functionality, offering opportunities to impart additional desirable properties such as improved mechanical strength, enhanced biocompatibility, controlled drug release, and responsiveness to external stimuli [12]. Nanocomposites synthesized from cellulose derivatives hold promise for applications such as scaffolds, wound dressings, drug delivery systems, and medical implants [13,14,15,16,17,18].

Yttrium oxide nanoparticles (Y_2_O_3_ NPs) are solid particles with a size ranging from 1–100 nm and have great attention due to their high surface reactivity, biocompatibility, and their inability to affect cellular viability [19].

The addition of Y_2_O_3_ NPs loaded to a self-assembled peptide (lauric acid-LLys-DPhe-LLys-NH_2_) conjugate nano-gel composite showed antibacterial properties against both Gram-positive and Gram-negative bacteria. In addition, nano-gel is considered a highly effective multifunctional biomaterial for wound healing without the exogenous delivery of growth factors [20].

This research aims to explore the synthesis of cellulose-based hydrogel-nano composites tailored for medical applications. By exploiting the inherent properties of cellulose and the unique attributes of yttrium oxide nanoparticles with a low concentration due to cytotoxicity [21], novel biomaterials with enhanced performance and functionality can be developed, addressing critical needs in modern healthcare. The envisaged prepared dried hydrogel can be used as a coating for medical devices, such as stents, and can be employed as dental chips for treating oral cavity infections such as periodontitis.

## 2. Materials and Methods

### 2.1. Materials

Microcrystalline Cellulose (MC), extra pure Avicel 101~50 μm particle size, pH102 crystalline type, white or almost white powder. Carboxymethyl cellulose sodium salt (CMC-Na) extra pure 99% with a molecular weight of 90,000 g/mol, degree of Substitution (DS) values range from 0.4 to 1.4, White to light yellow powder to crystal, high-viscosity ranges from 800 to 3100 mPas in a 2% solution at 25 °C. Citric acid anhydrous extra pure 99% was purchased from AZ chem. For chemicals (China), yttrium oxide powder (Y_2_O_3_), 99.9%, with an average size of 50–80 nm, was purchased from Hongwu Nano (Guangzhou, China).

### 2.2. Preparation of Hydrogel

Carboxymethyl cellulose sodium salt (CMC-Na) polymer (13% *w*/*w*, 0.02 mmol) and microcrystalline cellulose polymer (30% *w*/*w*, 8.1 mmol) were dispersed separately in 50 mL distilled water with continuous stirring using an overhead stirrer (500 rpm, VELP SCIENTIFICA, Usmate Velate, Italy) under ambient conditions until complete dissolution was observed. Microcrystalline cellulose suspension was then added to carboxymethyl cellulose solution with the same stirring conditions, and (13% *w*/*w*, 7.8 mmol) citric acid was added with continuous stirring (700 rpm at 50–60 °C) in a water bath for 4–6 h, glycerol (43% *w*/*w*, 54.3 mmol) was added to the mixture as a plasticizer. The solution was ultrasonicated to remove the produced air bubbles for 5 min. The mixture was poured into a 90 × 15 Petri dish and left to dry overnight at room temperature to form CMC-MC dry hydrogels.

### 2.3. Y_2_O_3_ NPs Loaded to Hydrogels

Suspension of Y_2_O_3_ NPs was loaded to the hydrogel during the mixing step, combining carboxymethyl cellulose sodium polymer with suspension microcrystalline cellulose for four different ratios (0.00% (0 mmol), 0.03% (0.017 mmol), 0.07% (0.04 mmol) and 0.10% (0.44 mmol)) to obtain hydrogels U1, U2, U3 and U4, respectively (MC-CMC/Y_2_O_3_ NPs).

### 2.4. Fourier Transform Infrared Spectroscopy (FTIR)

Fourier transform infrared spectroscopy (FTIR) spectra (4000 to 400 cm^−1^, 4 cm^−1^ spectral resolution) were measured using a PerkinElmer FT-IR (model SP2 spectrometer, Shelton, CT, USA) with an ATR attachment for detecting the chemical synthesis and properties of the hydrogels in their dry form.

### 2.5. X-ray Powder Diffraction

The dried film was subjected to X-ray powder diffraction (XRD) with 2θ angle range of 10–60° and a step angle of 0.02°, using a Malvern Panalytical-Model Aeris Research spectrometer (Almelo, The Netherlands).

### 2.6. Morphology

The surface morphology of the dried hydrogel was investigated using a scanning electron microscope (SEM). A cross-section of the dried hydrogels was examined under low vacuum to avoid the sputter coating, using FEI scanning electron microscopy model Versa 3D (Eindhoven, The Netherlands). 

### 2.7. Antimicrobial Activity

Antimicrobial activity was tested against Gram-negative bacteria (*E. coli*) and Gram-positive bacteria (*S. aureus*) according to the Kirby–Bauer method [17]. Inocula from overnight cultures were optically adjusted to obtain 1 × 10^8^ CFU/mL. Nutrient agar plates or Petri dishes were inoculated with 100 μL bacterial suspension, followed by placement of formulation-loaded hydrogel discs (5 mm diameter, n = 4) on the cultured agar plates. After 15 min, the *E. coli* cultured agar plates and *S. aureus* cultured agar plates were incubated at 37 °C for 48 h. Blank hydrogels in the dry form, which is U1 devoid of Y_2_O_3_ NPs, were also tested for their antimicrobial activity.

### 2.8. Determination of Ultimate Tensile Strength, Young’s Modulus, and Percent of Elongation at Break

The estimation of U.T.S. (Ultimate tensile strength), elasticity modulus, E, and percent of elongation at break were calculated by employing a TA-XT plus texture analyzer from stable Microsystems (Goldaming, Surrey, UK). The preparation of the sample involves cutting the hydrogels (in their dried form) into rectangular shapes (dimensions 1 × 3 cm). A caliper was used to determine each sample’s thickness and width, and the samples were subsequently fixed between the static (lower) and moveable (upper) grips. The speed of the test was set to 0.5 mm/s, and the contact force was 0.049 N. The maximum force before rupture and the cross-sectional area of the film sample were employed to calculate the U.T.S, Equation (1). Elongation percentage (%) before the cracking of each sample was determined according to the stress-strain plot, Equation (2). Young’s modulus was determined from stress over strain. Five replicates for each hydrogel were employed.

U.T.S = F_max_/A (1)

where:

- F_max_ is the maximum force (N) needed to pull the sample apart.
- A is the cross-sectional area (mm^2^), A = thickness × width.

Elongation% = (ΔL/L_i_) × 100 = (L_i_ − L)/L_i_ × 100 (2)

where:

- L_i_ is the initial length of the film sample.
- L is the film elongation at the moment of rupture.

### 2.9. Measurement of Friction Coefficient

The friction coefficient for samples U1–U4 was calculated using data obtained from the TA-XT Texture Analyzer. The protocol was conducted as described by the manufacturer. Horizontal friction testing was set up as described. The samples in the dried form were fixed onto the surface of the test plate and adhered to it. A standard metal piece (200 g provided by the manufacturer) was placed on the polymeric film, with the rough side of the metal facing the polymeric film. The rate of the metal piece movement was 5 mm/sec. The friction coefficient was calculated using Equation (3) [20].

Friction coefficient = friction force (g)/normal force (g)(3)

where the friction force is the average force resulting from the force-time curve, and the normal force is the applied weight of the standard metal piece.

### 2.10. Swelling Percentage

To study the water absorbance of samples U1–U4 with different Y_2_O_3_ NPs concentrations, pre-weighed (m_0_ 1 × 1 cm^2^) dried hydrogels were placed in 20 mL distilled water at room temperature for 24 h. At the end of the sampling time, the swollen hydrogel was removed from the media, wiped gently with filter paper to remove any excess water, and weighed (mt). The percentage of swelling was calculated using Equation (4), where m_0_ is the initial mass of dried hydrogel (g) and mt is the mass of swollen hydrogel (g) [17,18].

Swelling = (m_t_ − m_0_/m_0_) × 100% (4)

## 3. Results and Discussion

### 3.1. Formation Mechanism

**Scheme 1:**
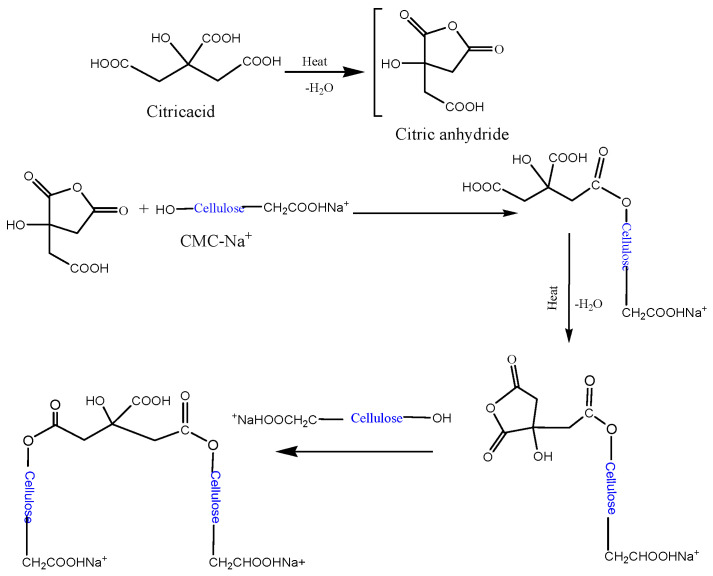
The envisaged mechanism of the cross-linking reaction between cellulose and CMC-Na^+^ by citric acid.

The formation of a hydrogel containing MC, CMC, and citric acid involves a combination of physical and chemical interactions (Scheme 1). Physical interactions are hydrogen bonding. All three components possess hydroxyl (-OH) groups, allowing for extensive hydrogen bonding between MC, CMC, and water molecules. This network of hydrogen bonds contributes significantly to the stability and structure of hydrogel. Moreover, the formation of weak attractive forces, known as Van der Waals forces, is possible between molecules due to temporary electronic fluctuations and is anticipated to contribute to the physical entanglement within the hydrogel network. Chemical interactions are primarily presented by esterification. Citric acid, a tribasic carboxylic acid, can react with the hydroxyl groups of both MC and CMC, forming an ester linkage. This cross-linking mechanism enhances the stability and structural integrity of the hydrogel [13,14]. The formation of the designated hydrogel relies on a synergistic combination of physical and chemical interactions. Optimizing the processing conditions and component characteristics can tailor the hydrogel properties for various applications.

### 3.2. FTIR Spectrum

**Figure 1:**
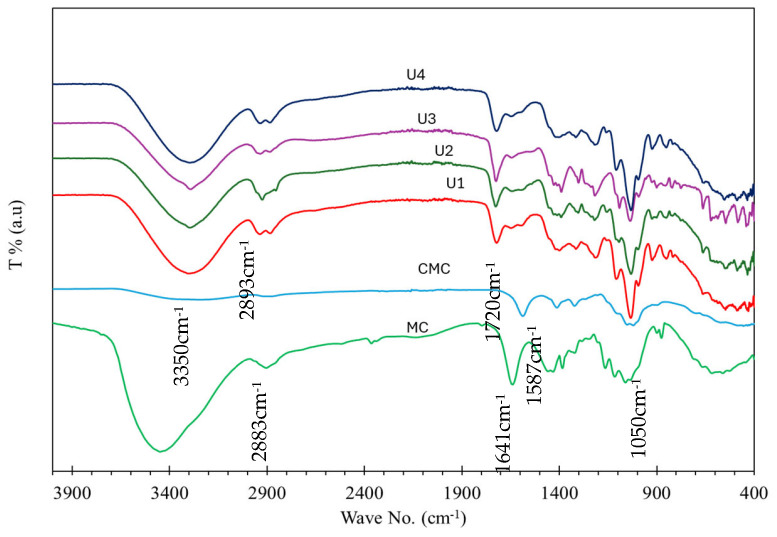
FT-IR spectra of U1–U4 hydrogels, CMC, and MC.

The FTIR spectra in Figure 1 indicate a similar composition for all samples. A strong band appearing at 3350 cm^−1^ is assigned to OH stretching. The bands at 2883 cm^−1^ and 2893 cm^−1^ are assigned to C-H stretching, while the band at 1720 cm^−1^, which is not found in CMC or CM, is assigned to C=O ester as proof of polymer formation. Weak bands located at 1600–1400 cm^−1^ are assigned to O-H carboxylic acid, and bands in the range 1250–1050 cm^−1^ are assigned to C-O-C stretching. The band at 1040–1050 cm^−1^ is assigned to CO-O-CO stretching anhydride. No bands for NPs were detected due to the very low amount of Y_2_O_3_ NPs loaded into all samples.

### 3.3. X-ray Powder Diffraction (XRD)

**Figure 2:**
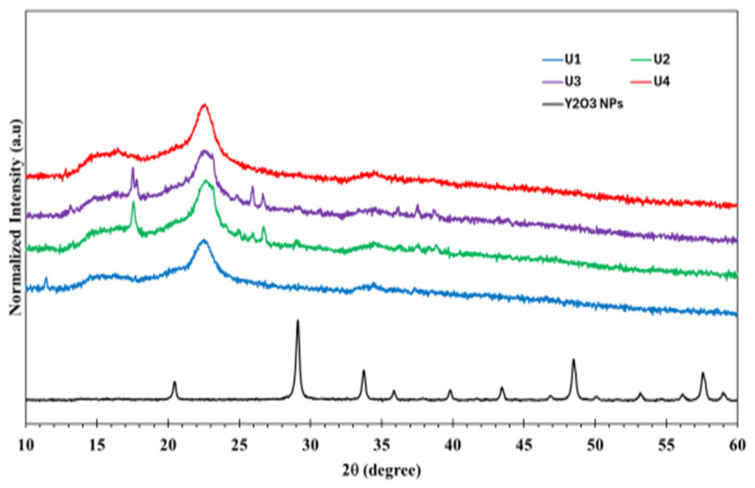
XRD for U1–U4 hydrogels in dry forms and the pure Y_2_O_3_NPs.

According to X-ray powder diffraction in Figure 2, the diffraction pattern for cellulose hydrogel loaded with different amounts of Y_2_O_3_ NPs, using high score plus software (version 5.2) for structural and profile fitting analysis, showed a cubic structure for the pure NPs with a lattice parameter a = 10.6122 + 0.0002 Å, the goodness of fit is 1.045, and the average grain size is 67.8 + 1.3 nm. However, the samples (U1–U4) displayed an amorphous structure, and some peaks were detected at 2θ 17.5°, 17.8°, 24.9°, 25.9°, 36.2°, and 37.6° which belongs to the crystalline sodium citrate that form by reaction of excess citric acid and CMC-Na^+^ salt. No peaks belonging to Y_2_O_3_ NPs were detected, as the amount of loaded Y_2_O_3_ NPs is below the detection limit.

### 3.4. Antimicrobial Activity

The results of the antimicrobial activity of dried hydrogel samples U1–U4 with different Y_2_O_3_ NPs loading against *S. aureus* and *E. coli* are presented in Table 1. The results of the zone of inhibition (ZOI) reveal that the largest inhibition was related to a high concentration of Y_2_O_3_ NPs. Furthermore, U1, the hydrogel devoid of nanoparticles, seems to possess antimicrobial activity. The crosslinked polymer showed a respectable antimicrobial effect. The antibacterial activity of the polymeric hydrogel could be attributed to the generated charge or alterations in the microenvironmental pH, which provides a hostile medium for microbial growth [22]. The observed antimicrobial activity against two different species, which are Gram-positive aureus and Gram-negative coli, indicates the versatile applications in vivo, as the disruptions of normal flora in the oral cavity, skin, or medical devices may face the growth of different strains and species. This will highlight the significance of continuously developing new materials to defend the prevalence of infectious diseases.

### 3.5. Morphology

**Figure 3:**
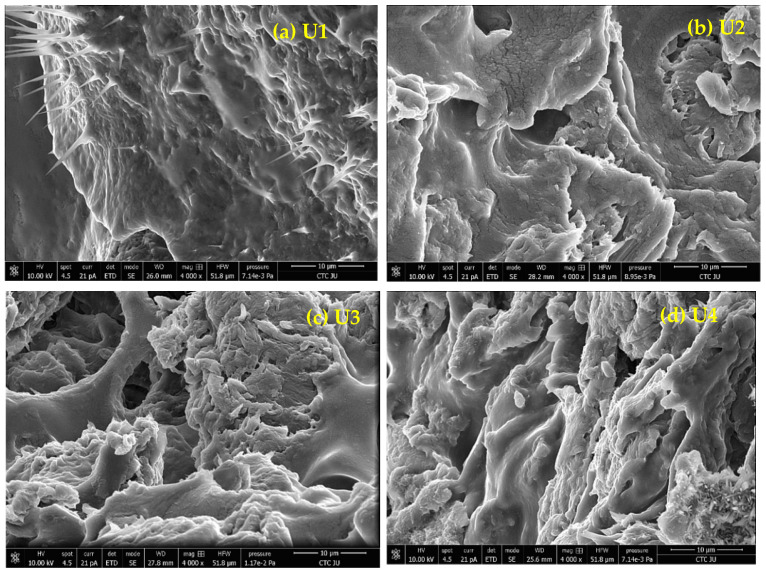
SEM micrographs of MC-CMC/Y_2_O_3_ Nanocomposite (U1–U4).

SEM micrographs for samples MC-CMC/Y_2_O_3_ nanocomposite are shown in Figure 3a–d. The dried gels with Y_2_O_3_ NPs additions had a heterogeneous and uniform distribution. Interesting spikes were observed in U1 (U1: MC-CMC hydrogel devoid of nanoparticles). The spikes could be attributed to the unreacted citric acid and traces of CMC Na. However, the surface appeared smooth. The addition of nanoparticles appears to increase the roughness in the surface, as observed in Figure 3b–d representing the Y_2_O_3_ NPs addition with different ratios, where the roughness appears mostly in Figure 3d: U4. The observed SEM micrographs were consistent with the author’s observations in the laboratory. The micrographs show that the microstructural pores increase with increasing the nanoparticle load.

### 3.6. Mechanical Properties 

**Figure 4:**
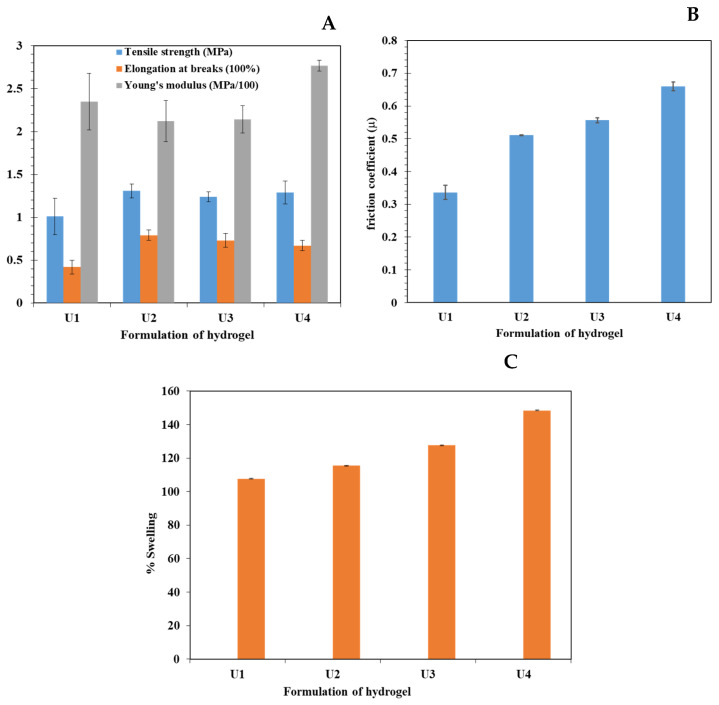
(**A**) UTS, Elongation at break and Young’s modulus. (**B**): Friction coefficient of hydrogels and (**C**) water uptake represented as % swelling.

The prepared MC-CMC Y_2_O_3_ nanocomposites were mechanically flexible and self-standing. The mechanical properties of the hydrogels are presented in Figure 4A. The addition of Y_2_O_3_ NPs did not affect the mechanical properties, where the U.T.S was similar in the tested formulations, and Young’s modulus was not significant in the tested formulations. Furthermore, the percentage of elongation was not affected by the amount of nanoparticles. These results suggest that the addition of Y_2_O_3_ NPs may not have a significant effect and would not contribute to the elasticity of the medical device and that the mechanical properties were attributed to the amount and type of involved polymers. 

### 3.7. Friction Coefficient 

The friction coefficient was found to be dependent on the ratio of nanoparticles. Increased nanoparticle concentration led to an increase in the friction coefficient during the crosslinking reaction of nanoparticles, as observed in Figure 4B. Upon revising the micrographs of the prepared hydrogels, it is assumed that the spikes were fragile and could be crushed and shattered into small particles upon loading the metal piece. This facilitates the movement of the metal piece and lowers the friction coefficient value (as observed in Figure 4B). In addition, increasing the nanoparticle load led to an increased resistance to movement and a higher friction coefficient. The medical hydrogels are anticipated to reduce microbial infection in order to increase the lifespan of the medical device and thus reduce the burden on the medical team and lower the suffering of the patient attributed to the resultant pain of the medical procedures. This pain is attributed to the generated friction between the delicate surface of the living tissues and the surface of the medical device. The designated friction not only generates pain but also causes trauma to the delicate tissues. The production of low-friction hydrogel is deemed appropriate to ease the pain of the patient [20].

### 3.8. Swelling

Figure 4C illustrates the percentage swelling of the prepared hydrogels where a higher concentration of nanoparticles showed a higher percentage of swelling. This suggests that the higher concentration of loaded nanoparticles swell at a higher rate after 24 h by using water pH = (6.5–7). The results of water uptake are compliant with the SEM micrographs, where increasing the porosity of the hydrogel shall increase the absorbed water to be entangled in the formed pores.

## 4. Conclusions

Cellulose hydrogel based on microcrystalline cellulose MC and carboxymethyl cellulose CMC was successfully synthesized. The mechanism of formation was a crosslinking reaction where citric acid was the cross-linker. The prepared cellulose derivative hydrogels were loaded with Y_2_O_3_ nanoparticles during the preparation of hydrogel. The addition of Y_2_O_3_ nanoparticles to the cellulose hydrogel showed antibacterial activity. This study considered the trauma of living tissue and studied friction, which was acceptable for in vivo use in tissue. However, it increased with a higher load of Y_2_O_3_. The hydrogel has a porous morphology with an increased ratio of Y_2_O_3_ and showed the highest swelling percentage of 150%. The obtained results showed the feasibility of the implementation of our dried hydrogels in in vivo applications, where they may be employed as a coating for medical devices or implemented as a drug delivery carrier for intra-dental pockets and chips or wound dressings.

## Figures and Tables

**Table 1:** The zone of inhibition (ZOI) effect of U1–U4 samples tested on two different species, *S. aureus* and *E. coli*.

Sample	*S. auras* ZOI (mm)	*E. coli* ZOI (mm)
U1	15.0 ± 0.1	12.0 ± 0.2
U2	20.7 ± 0.1	18.0 ± 0.1
U3	20.5 ± 0.2	20.7 ± 0.1
U4	20.1 ± 0.1	20.5 ± 0.1

## Data Availability

The original contributions presented in the study are included in the article, further inquiries can be directed to the corresponding author.

## References

[1] Annabi N., Zhang Y.N., Assmann A., Sani E.S., Cheng G., Lassaletta A.D., Vegh A., Dehghani B., Ruiz-Esparza G.U., Wang X. (2017). Engineering a highly elastic human protein-based sealant for surgical applications. Sci. Transl. Med..

[2] Varaprasad K., Raghavendra G.M., Jayaramudu T., Yallapu M.M., Sadiku R. (2017). A mini review on hydrogels classification and recent developments in miscellaneous applications. Mater. Sci. Eng. C.

[3] Gaharwar A.K., Peppas N.A., Khademhosseini A. (2014). Nanocomposite hydrogels for biomedical applications. Biotechnol. Bioeng..

[4] Dacrory S., Abou-Yousef H., Abou-Zeid R.E., Kamel S., Abdel-Aziz M.S., Elbadry M. (2018). Preparation and characterization of eco-friendly carboxymethyl cellulose antimicrobial nanocomposite hydrogels. J. Renew. Mater..

[5] Klemm D., Heublein B., Fink H.P., Bohn A. (2005). Cellulose: Fascinating biopolymer and sustainable raw material. Angew. Chem. Int. Ed..

[6] Wang J., Sun Y., Liu X., Kang Y., Cao W., Ye J., Gao C. (2024). An antibacterial and anti-oxidant hydrogel containing hyperbranched poly-L-lysine and tea polyphenols accelerates healing of infected wound. Biomater. Adv..

[7] Chang C., Zhang L. (2011). Cellulose-based hydrogels: Present status and application prospects. Carbohydr. Polym..

[8] Tarawneh O., Hammad A.M., Mahfouz H.A., Hamadneh L., Hamed R., Hamadneh I., Al-Assi A.R. (2023). Development of Mucoadhesive Cellulose Derivatives Based Films for the Treatment of Vaginal Candidiasis. Cellul. Chem. Technol..

[9] Tarawneh O., Hamadneh I., Huwaitat R., Al-Assi A.R., El Madani A. (2021). Characterization of Chlorhexidine-Impregnated Cellulose-Based Hydrogel Films Intended for the Treatment of Periodontitis. BioMed Res. Int..

[10] Nezhad-Mokhtari P., Akrami-Hasan-Kohal M., Ghorbani M. (2020). An injectable chitosan-based hydrogel scaffold containing gold nanoparticles for tissue engineering applications. Int. J. Biol. Macromol..

[11] Mahmoud N.N., Hikmat S., Ghith D.A., Hajeer M., Hamadneh L., Qattan D., Khalil E.A. (2019). Gold nanoparticles loaded into polymeric hydrogel for wound healing in rats: Effect of nanoparticles’ shape and surface modification. Int. J. Pharm..

[12] El Achaby M., El Miri N., Hannache H., Gmouh S., Ben Youcef H., Aboulkas A. (2018). Production of cellulose nanocrystals from vine shoots and their use for the development of nanocomposite materials. Int. J. Biol. Macromol..

[13] Janmohammadi M., Nazemi Z., Salehi A., Seyfoori A., John J.V., Nourbakhsh M.S., Akbari M. (2023). Cellulose-based composite scaffolds for bone tissue engineering and localized drug delivery. Bioact. Mater..

[14] Courtenay J.C., Johns M.A., Gale Beck F., Deneke C., Lanzoni E.M., Costa C.A., Scott J.L., Sharma R.I. (2017). Surface-modified cellulose scaffolds for tissue engineering. Cellulose.

[15] Tarawneh O., Al-Assi A.R., Hamed R., Sunoqrot S., Hasan L., Al-Sheikh I., Al-Qirim R., Alhusban A.A., Naser W. (2019). Development and characterization of k-carrageenan platforms as periodontal intra-pocket films Trop. J. Pharm. Res..

[16] Ahmed A.Y., Ayad D.M., Youssef A.M., Abdelaal M.Y. (2024). Preparation and Evaluation of PANI/PVA/Cu Bionanocomposite Hydrogel via Green Route. Egypt. J. Chem..

[17] Esser V.M., Elefson D.E. (1970). Experiences with the Kirby-Bauer method of antibiotic susceptibility testing. Am. J. Clin. Pathol..

[18] Salmon J.H., Rat A.C., Sellam J., Michel M., Eschard J.P., Guillemin F., Jolly D., Fautrel B. (2016). Economic impact of lower-limb osteoarthritis worldwide: A systematic review of cost-of-illness studies. Osteoarthr. Cartil..

[19] Akhtar M.J., Ahamed M., Alrokayan S.A., Ramamoorthy M.M., Alaizeri Z.M. (2020). High Surface Reactivity and Biocompatibility of Y_2_O_3_ NPs in Human MCF-7 Epithelial and HT-1080 Fibro-Blast Cells. Molecules.

[20] Chawla V., Sharma S., Singh Y. (2023). Yttrium Oxide Nanoparticle-Loaded, Self-Assembled Peptide Gel with Antibacterial, Anti-Inflammatory, and Proangiogenic Properties for Wound Healing. ACS Biomater. Sci. Eng..

[21] Moriyama A., Takahashi U., Mizuno Y., Takahashi J., Horie M., Iwahashi H. (2019). The truth of toxicity caused by yttrium oxide nanoparticles to yeast cells. J. Nanosci. Nanotechnol..

[22] Haktaniyan M., Bradley M. (2022). Polymers showing intrinsic antimicrobial activity. Chem. Soc. Rev..

